# *Ocimum gratissimum* Linn. Leaves reduce the key enzymes activities relevant to erectile dysfunction in isolated penile and testicular tissues of rats

**DOI:** 10.1186/s12906-019-2481-0

**Published:** 2019-03-19

**Authors:** Oluwafemi Adeleke Ojo, Adebola Busola Ojo, Babatunji Emmanuel Oyinloye, Basiru Olaitan Ajiboye, Omosola Olufisayo Anifowose, Ayodeji Akawa, Oluranti Esther Olaiya, Oluwaseun Ruth Olasehinde, Abidemi Paul Kappo

**Affiliations:** 1 Department of Biochemistry, Afe Babalola University, Ado-Ekiti, Nigeria; 2 Department of Biochemistry, University of Ilorin, Ilorin, Nigeria; 3 Department of Biochemistry, Ekiti State University, Ado-Ekiti, Nigeria; 4 Department of Physiology, Afe Babalola University, Ado-Ekiti, Nigeria; 5 Department of Medical Biochemistry, Afe Babalola University, Ado-Ekiti, Nigeria; 6 Biotechnology and Structural Biology (BSB) Group, Department of Biochemistry and Microbiology, University of Zululand, KwaDlangezwa, 3886 South Africa

**Keywords:** Erectile dysfunction, Angiotensin I –converting enzyme (ACE), Phosphodiesterase-5 (PDE-5), Acetylcholinesterase (AChE), Arginase, *Ocimum gratissimum*

## Abstract

**Background:**

*Ocimum gratissimum* L. is a medicinal plant widely grown in tropical and subtropical regions with the leaf decoction usually taken in folk medicine to enhance erectile performance in men although the probable mechanism of actions remains undetermined. This study examined the inhibitory potentials of *Ocimum gratissimum* leaves on some key enzymes associated with erectile dysfunction in penile and testicular tissues of the rat.

**Methods:**

Inhibitory effect of aqueous extract (1:10 *w*/*v*) of *O. gratissimum* leaves on the activities of phosphodiesterase-5 (PDE-5), arginase, angiotensin I –converting enzyme (ACE), and acetylcholinesterase (AChE) in penile and testicular tissues were assessed. Also, the extract was investigated for ferric reducing antioxidant property(FRAP) and 1,1-diphenyl-2-picryl-hydrazil (DPPH) radical scavenging abilities.

**Results:**

The extract showed higher PDE-5 (IC_50_ = 43.19 μg/mL), ACE (IC_50_ = 44.23 μg/mL), AChE (IC_50_ = 55.51 μg/mL) and arginase (IC_50_ = 46.12 μg/mL) inhibitory activity in the penile tissue than PDE-5 (IC_50_ = 44.67 μg/mL), ACE (IC_50_ = 53.99 μg/mL), AChE (IC_50_ = 60.03 μg/mL) and arginase (IC_50_ = 49.12 μg/mL) inhibitory activity in the testicular tissue homogenate. Furthermore, the extract scavenged free radicals and in a dose-dependent manner.

**Conclusion:**

The enzyme activities displayed might be associated with the bioactive compounds present in the extract which could possibly explain its use in the management of erectile dysfunction (ED).

## Background

The occurrence of erection involves the neuronal, vascular, endocrine and local secretory interplay. The processes involved are blood vessel dilation, trabecular smooth muscle relaxation, and activation of the veno-occlusive mechanism of the corpus carvenosum and spongiosum tissues [1]. Erectile dysfunction (ED) is the inability to achieve and maintain penile tumescence adequate for the suitable erotic act [2]. It occurs as a result of the decrease in bioavailability of nitric oxide (NO) due to decreased expression of endothelial tissue nitric oxide synthase (NOS) activity and/or increased elimination of NO [3]. However, NOS and arginase vie for L-arginine as a substrate in cells. In the urea cycle, L-arginine is converted to ornithine and urea and the conversion is catalyzed by the enzyme arginase [4]. The fast hydrolysis of secondary messengers such as cyclic adenosine monophosphate (cAMP) and cyclic guanosine monophosphate (cGMP) have been linked to pathologies like ED [5]. Cyclic nucleotide phosphodiesterase (PDE) enzymes regulate the cytosolic levels of secondary messengers [4, 6]. Predominantly in the smooth muscle of corpora cavernosum is phosphodiesterase type-5. Hence, the mitigation of phosphodiesterase-5 (PDE-5) can also be a therapeutic approach in the management of ED [6]. Additionally, acetylcholinesterase (AChE) inhibition is an alternative technique for managing ED because it controls the levels of acetylcholine [7]. Moreover, the increasing AChE activity decreases the level of acetylcholine on conversion to its products acetate and choline [8, 9]. Earlier studies reported that inhibition of AChE can increase the level of acetylcholine thereby improving erection [10, 11]. Moreover, the renin–angiotensin system (RAS) is also a key aspect in the development of erectile dysfunction. Increase in the angiotensin-I converting enzyme (ACE) caused by erectile dysfunction simultaneously leads to the generation of angiotensin-II [12–14]. Hence, ACE activity inhibition leads to an increase in erectile performance and a significant reduction of angiotensin-II levels in patients with ED [15]. Oxidative stress-induced ED occurs when reactive oxygen species such as superoxide anion (O_2_^−^) reacting with nitric oxide gas, forms peroxynitrite (ONOO_2_) and reduces the biological presence of NO [16]. Adequate intake of natural plant products may protect against oxidative stress and reduce the chance of developing several diseases like male sexual dysfunction [17]. Furthermore, the management of ED with conventional medicine is connected with adverse side effects and toxicities. Thus, the need to continuously explore medicinal plants with sexual enhancing properties may serve as a cheap and safe alternative therapy [18].

*Ocimum gratissimum* (OG) is a perennial herb that belongs to the family Lamiaceae. It is thought to originate from Asia and Africa [19]. In Nigeria and other parts of the world, it is used as a traditional vegetable condiment and oral care products. Furthermore, OG had been shown to possess numerous pharmacological properties hence its use in traditional or alternative medicine. These properties include antioxidant [20–24]; anti-anemic [25]; antidiarrhoeal [26] and protective effects on hepato-renal indices [27]. In a study conducted by Iweala and Obidoa [29], using *Ocimum gratissimum*-supplemented diet (OGSD), rats fed with OGSD for 6 months showed an increased number of spermatozoa suggesting the capacity of OG to enhance reproductive ability [28]. Another study conducted by Pande and Pathak [29] revealed that ethanolic extract of OG, significantly increased the sexual behaviors of normal male albino mice at a dose of 100, 250, and 500 mg/kg body weight for 7 days with 500 mg/kg having the most noticeable effect, without any gastric ulceration and adverse effects [29, 30]. In addition to this, Ebong et al. [31] reported that combined extracts of *Moringa oleifera* and *Ocimum gratissimum* had better ameliorative effects on testicular architecture and spermatogenesis after 28 days of treatment than *Moringa* extract alone in diabetic rats. According to the authors, a combination of the two plant extracts thus provides a cheap alternative to treating diabetes-associated testicular damage and sexual dysfunction [31]. The plant leaf extract is used in alternative medicine for the management of erectile dysfunction [31]. However, there is limited information on the possible mechanisms of actions of *O. gratissimum* on the penile and testicular function. Thus, this study investigated the inhibitory effects of aqueous extract from *O. gratissimum* leaves on enzymes such as (phosphodiesterase-5 (PDE-5), angiotensin-1 converting enzyme (ACE), acetylcholinesterase (AChE), and arginase) relevant to ED in penile and testicular tissue homogenates. Antioxidant activity (ferric reducing antioxidant property (FRAP) and 1,1-diphenyl-2-picryl-hydrazil (DPPH)) of the leaves were also examined.

## Methods

### Plant collection

*O. gratissimum* leaves were bought from a King’s market, Ado-Ekiti in November 2017. It was authenticated by Mr. Omotayo with herbarium number (UHAE 15) and deposited at the Department of Plant Science, Ekiti State University, Ado-Ekiti, Nigeria. The *Ocimum gratissimum* leaves were dried and pulverized using a blender. The powder obtained weighing 70 g was macerated in 700 mL of distilled water for 24 h at room temperature as described by [32]. It was then filtered and the resulting filtrate was concentrated on a steam bath to give a yield of 40.45 g of the residue.

### Chemicals and reagents

Chemicals such as acetylthiocholine iodide, 5,5′-dithio-bis (2- nitrobenzoic acid) (DTNB), thiobarbituric acid (TBA), trichloroacetic acid (TCA), and 1,1-diphenyl-2 picrylhydrazyl (DPPH) were sourced from Sigma-Aldrich, Chemie GmbH (Steinheim, Germany). Acetic acid was procured from BDH Chemical Ltd., (Poole, England). A Jenway UV-visible spectrophotometer (Model 6305; Jenway, Barlo World Scientific, Dunmow, United Kingdom) was used to measure absorbance.

### Handling of experimental animals

Twenty adult male Wistar albino rats (weighing between 290 ± 5 g) of about 10–14 weeks old were obtained from Animal breeding house of Afe Babalola University and five [5] rats were used for each of the assays carried out. They were handled in accordance with the guide for Care and Use of Laboratory Animals formulated by the National Academy of Science, issued by the National Institute of Health (USA). The ethical guidelines were followed in accordance with National and Institutional guidelines for the protection of animal welfare during the experiments. The study was approved by Afe Babalola University ethical review committee (ABUERC) with reference number 17/ABUAD/066. The rats were allowed to acclimatize for 2 weeks and maintained at room temperature under laboratory conditions (12 h light/dark cycle) with access to rat chow and water ad libitum.

### Preparation of penile and testicular tissue homogenate

Experiment animals were placed under ether anesthesia and euthanized by cervical dislocation, then tissue samples were collected. Penis and testes were removed, washed, bottled dry and weighed. These tissues were homogenized in cold saline (1/10 *w*/*v*) at approximately 1200 rpm in a homogenizer for 10 min at 3000 rpm. The supernatant was collected and used for the inhibitory enzyme activity assay.

### Phosphodiesterase-5 (PDE-5) inhibitory activity

The ability of the extracts to inhibit PDE-5 activity was evaluated by [33]. The assay solution containing 5 mM of the substrate (p-nitrophenyl phenyl phosphonate), 100 μl of tissue (penile and testicular) supernatant, 20 mM Tris-HCL (pH 8.0) and the aqueous extracts of *O. gratissimum* (20–100 μg/ml) were incubated at 37 °C for 10 min. The amount of p-nitrophenol produced was read as a change in absorbance after 5 min at 400 nm. The control experiment was performed without the extracts and sildenafil. PDE-5 inhibitory enzyme activity was expressed as percentage inhibition:

1 $$ PDE-5\ \mathrm{inhibition}\ \left(\%\right)=\left[\left({\mathrm{Abs}}_{\mathrm{control}}-{\mathrm{Abs}}_{\mathrm{samples}}\right)/{\mathrm{Abs}}_{\mathrm{control}}\right]\times 100 $$

where Abs _control_ is the absorbance without the extract and Abs _samples_ are the absorbance with extract.

### Arginase inhibitory activity

The penis and testicular homogenates were prepared by homogenizing 10 g (*w*/*v*) of penile and testicular tissue in cold buffer (phosphate buffer, pH 7.2). The homogenized tissue samples were centrifuged for 20 min at 4000 rpm and the supernatant was used as the source of enzyme. The activity of arginase was estimated by the amount of urea produced via Ehrlich’s reagent. The solution comprised in final volume 1.0 mM Tris-HCl buffer pH 9.5, 1.0 mM MnCl, 0.1 M arginine solution, an aqueous extract of *O. gratissimum* (20–100 μg/ml) /L-2-amino-[4-(20-hydroxyguanidino)] butyric acid (LNOHA) which was made up to 1.0 mL. The solution was incubated for 10 min at 37 °C and stopped by adding 2.5 mL Ehrlich reagent (2.0 g of p-dimethyl amino benzaldehyde in 20 mL of concentrated hydrochloric acid and made up to 100 mL with distilled water). Absorbance was read after 20 min at 450 nm. The control experiment was performed without the test sample or standard and arginase inhibitory activity was calculated as percentage inhibition [34].

### Acetylcholinesterase activity assay

Penile and testicular tissue samples were homogenized in cold phosphate buffer 0.1 M, pH 7.2 and were used as the source of AChE enzyme. The inhibitory effect of the aqueous extracts of *O*. *gratissmum* (20–100 μg/ml) and standard drug prostigmine on AChE activity was evaluated as described by [8] with slight modifications. AChE activity was estimated in a solution containing 200 μL tissue homogenate, 100 μL of 5,5-dithiol-bis (2-nitrobenzoic) acid (DTNB 3.3 mM, in 0.1 M phosphate buffered solution, pH 7.0, containing 6 mM NaHCO_3_), aqueous extracts of *O*. *gratissmum*. This was incubated for 20 min at 25 °C, and acetylthiocholine iodide was added. The absorbance of the enzyme activity was read at 412 nm. AChE activity was expressed as percentage inhibition [1].

### Angiotensin-I-converting enzyme (ACE) inhibitory activity

Inhibition of the ACE activity of aqueous extracts of *O*. *gratissmum* was determined according to the described protocol of [35]. Varying concentrations of the aqueous extract (20–100 μg/ml) and standard drug lisinopril and 50 *μ*L of homogenate from penile and testicular tissues as a source of ACE enzyme (4 mU/mL) were pre-incubated at 37 °C for 15 min. Thereafter, the enzymatic process was started by adding 200 *μ*L of 8.33 mM ACE substrate [hippuryl-l-histidyl-l-leucine (HHL)] in 125 mM of Tris-HCl buffer (pH 8.3) to the solution and incubated at 37 °C for 30 min. The reaction was terminated by adding 300 *μ*L of 1 M HCl. The hippuric acid (Bz-Gly) generated by the reaction was removed with 2 mL ethyl acetate and further centrifuged to separate the ethyl acetate layer, it was later transferred to a volumetric flask and evaporated to dryness. The obtained residue was reconstituted in distilled water and its absorbance was read at 228 nm. The control experiment was done without the aqueous extract or standard drug lisinopril. ACE percentage inhibition was then calculated in Eq. (1).

### Determination of antioxidant activities

#### Ferric reducing antioxidant ability

Reducing power of the aqueous extract was evaluated via reduction of the FeCl_3_ solution by [36]. 3 mL of the extract (20–100 μg/ml) was added to 3 mL of 200 mM sodium phosphate buffer (pH 6.6) and 3 mL of 1% potassium ferricyanide. The solution was incubated for 20 min at 50 °C in a water bath and 3 mL of 10% trichloroacetic acid was used to stop the reaction. The sample was then centrifuged at 650 g for 10 min and 5 mL of the supernatant was added to an equal volume of water and 1.0 mL, 0.1% FeCl_3_. Absorbance was read at 700 nm. Vitamin C was used as the standard for assessing reducing power and expressed as percentage inhibition.

### Free radical scavenging ability

Free radical scavenging ability of the aqueous extract against DPPH^.^ was assessed using the method of [37]. Concisely, suitable dilution of the aqueous extract (20–100 μg/ml) was added to 1 mL 0.4 mM DPPH solution in methanol. The solution was kept in the dark for 30 min and the absorbance was read at 516 nm. DPPH^*^ scavenging ability was estimated with reference to the control and the standard drug used for assessing DPPH activity was vitamin C.

$$ {\mathrm{DPPH}}^{\ast}\mathrm{scavenging}\ \mathrm{ability}\ \left(\%\right)=\left[\left({\mathrm{Abs}}_{\mathrm{control}}-{\mathrm{Abs}}_{\mathrm{samples}}\right)/{\mathrm{Abs}}_{\mathrm{control}}\right]\times 100 $$

### Data analysis

Data generated were analyzed by one-way analysis of variance (SPSS, Version 20.0, IBM Corporation, NY, USA) one-way ANOVA using Duncan multiple range *posthoc* test (DMRT). Results are presented as the mean ± SD in triplicates (*n* = 3). Values were considered to be significantly different at *p* < 0.05.

## Results

**Fig. 1:**
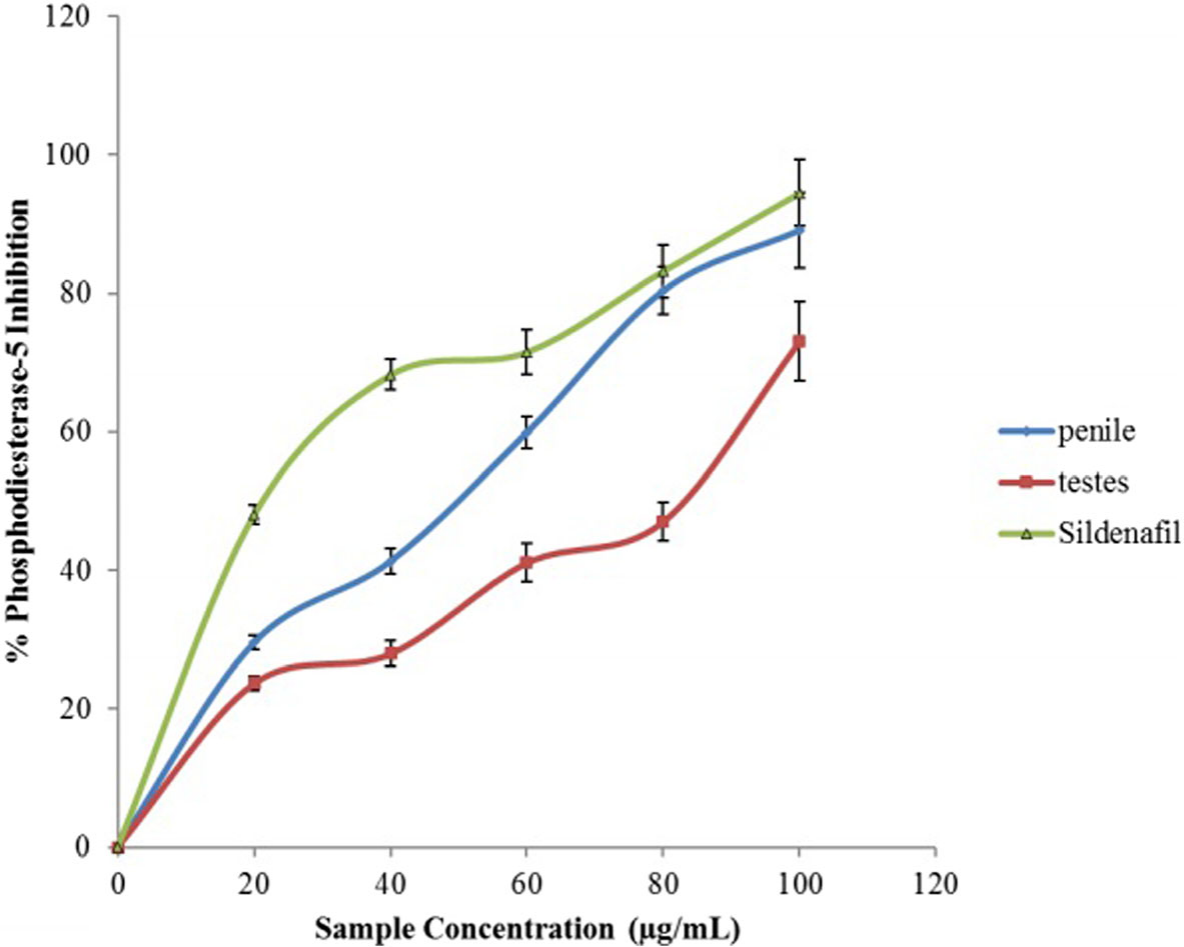
Inhibition of Phosphodiesterase-5 activity in rats’ genitals by aqueous extract from *Ocimum gratissimum* leaves

Phosphodiesterase-5 activities (PDE-5) of aqueous extract of *O*. *gratissmum* in penile and testicular tissue homogenates were evaluated and presented in Fig. 1. The result showed that the aqueous extract of *O*. *gratissmum* inhibited PDE-5 enzymes. Taking into consideration the IC_50_ (lower IC_50_ value means stronger enzyme inhibition) presented in Table 1, it showed that the extract had a greater inhibitory activity on penile phosphodiesterase-5 (IC_50_ = 43.19 μg/mL) than testicular phosphodiesterase-5 (IC_50_ = 44.67 μg/mL). Though, sildenafil had the highest activity as shown by the IC_50_ (26.65 μg/mL).

**Table 1:** IC_50_ values (μg/mL) of ferric reducing power, DPPH, PDE-5, arginase, ACE and AChE inhibitory activities in rat penile and testicular tissue by *O. gratissimum* leaf extracts and standard drugs/inhibitors

Sample	PDE-5 (μg/mL)	Arginase (μg/mL)	ACE (μg/mL)	AChE (μg/mL)	FRAP (μg/mL)	DPPH (μg/mL)
Penis	43.19 ± 0.02^c^	46.12 ± 0.15^c^	44.23 ± 0.71^a^	55.51 ± 0.45^a^	–	–
Testes	44.67 ± 0.01^b^	49.12 ± 0.12^b^	53.99 ± 0.85^a^	60.03 ± 0.34^a^	–	–
Sildenafil (μg/mL)	26.65 ± 0.10^a^	–	–	–	–	–
L-NOHA (μg/mL)	–	42.48 ± 0.21^a^	–	–	–	–
Lisinopril (μg/mL)	–	–	38.34 ± 0.01^a^	–	–	–
Prostigmine (μg/mL)	–	–	–	48.01 ± 1.02^a^	–	–
Vitamin C (μg/mL)	–	–	–	–	46.16 ± 0.51^a^	53.81 ± 0.02^a^
*O. gratissimum* extract	–	–	–	–	56.55 ± 0.78^a^	69.41 ± 0.01^a^

Values represent mean ± standard deviation (*n* = 3). Values with the same superscript along the column are not significant (*p* < 0.05) different. Sildenafil^*^: standard drug for phosphodiesterase-5 (PDE-5); L-NOHA^*^: standard inhibitor for arginase; prostigmine^*^: standard drugs for AChE; Lisinopril^*^: standard drug for ACE; Vitamin C^*^: standard drug for ferric reducing power (FRAP) and DPPH

**Fig. 2:**
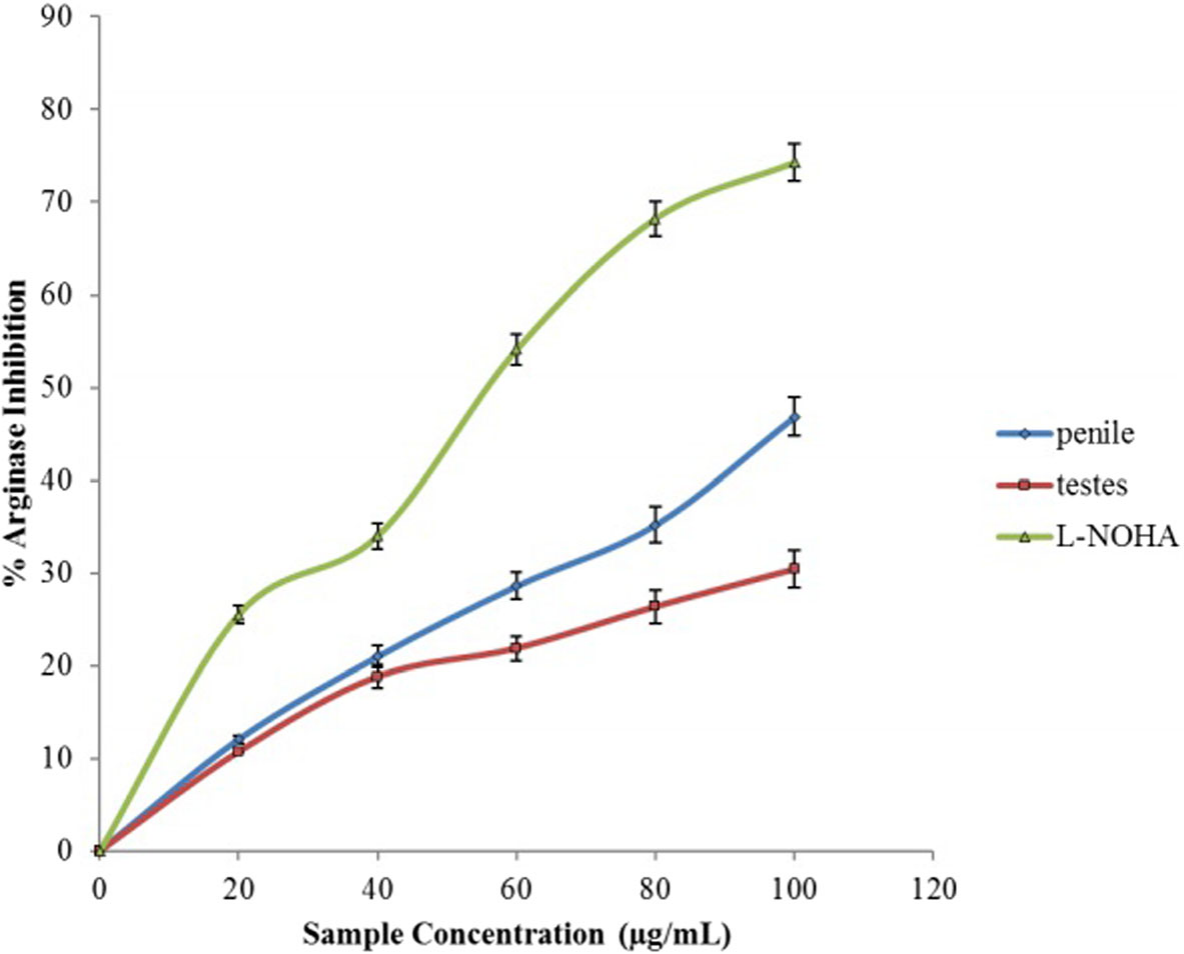
Inhibition of Arginase activity in rats’ genitals by aqueous extract from *Ocimum gratissimum* leaves

Arginase inhibitory activity by the aqueous extract of *O*. *gratissmum* is displayed in Fig. 2. The extract inhibited arginase activity in a concentration-dependent manner. Though, the IC_50_ in Table 1 showed that the aqueous extract of *O*. *gratissmum* had a greater inhibitory activity on penile arginase (46.12 μg/mL) than testicular arginase (49.12 μg/ mL). Though, L-NOHA had the highest inhibitory activity as shown by the IC_50_ (42.48 μg/mL).

**Fig. 3:**
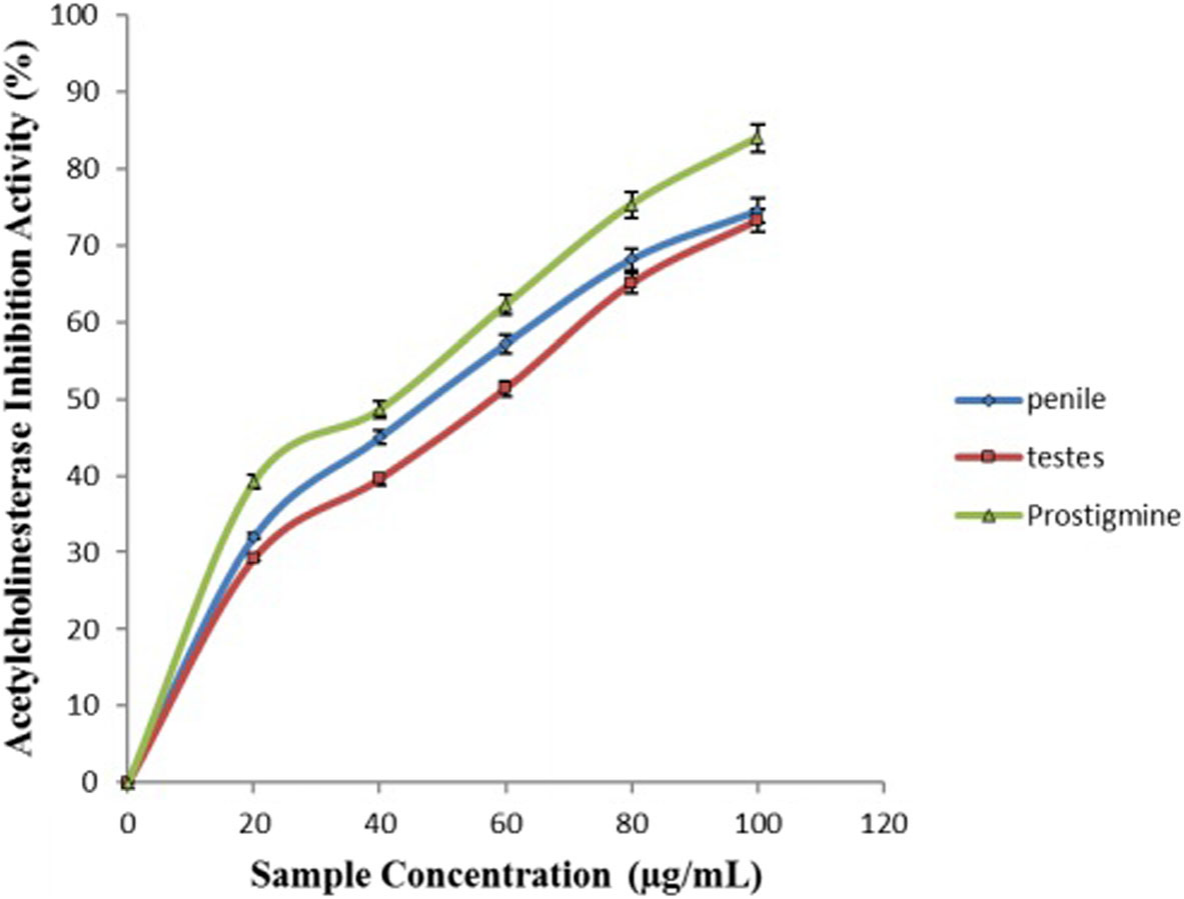
Inhibition of Acetylcholinesterase activity in rats’ genitals by aqueous extract from *Ocimum gratissimum* leaves

Figure 3 showed the result of the inhibitory activity of aqueous extract of *O*. *gratissmum* on acetylcholinesterase (AChE) enzyme. AChE activity was inhibited by aqueous extract of *O*. *gratissmum* as the concentration increases. Hence, aqueous extract of *O*. *gratissmum* had a better inhibitory activity on penile AChE activity (IC_50_ = 55.51 μg/mL) than testicular AChE activity (IC_50_ = 60.03 μg/mL) as shown by their IC_50_ values listed in Table 1.

**Fig. 4:**
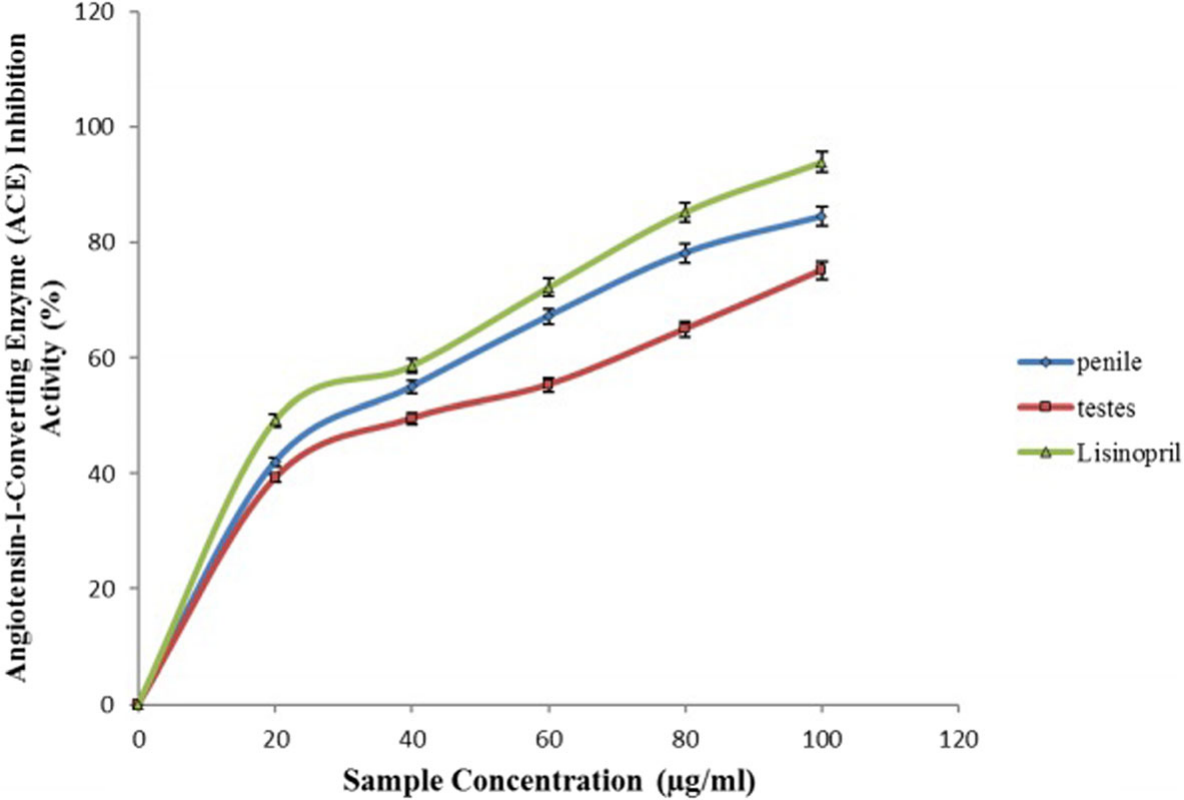
Inhibition of Angiotensin-I-converting enzyme activity in rats’ genitals by aqueous extract from *Ocimum gratissimum* leaves

Figure 4 revealed the inhibition of ACE activity by aqueous extract of *O*. *gratissmum* in penile and testicular tissue homogenates. ACE activity was inhibited by both the aqueous extract of *O*. *gratissmum* and lisinopril. However, aqueous extract of *O*. *gratissmum* had a better inhibitory activity on penile than testicular ACE activity as shown by their IC_50_ values listed in Table 1.

**Fig. 5:**
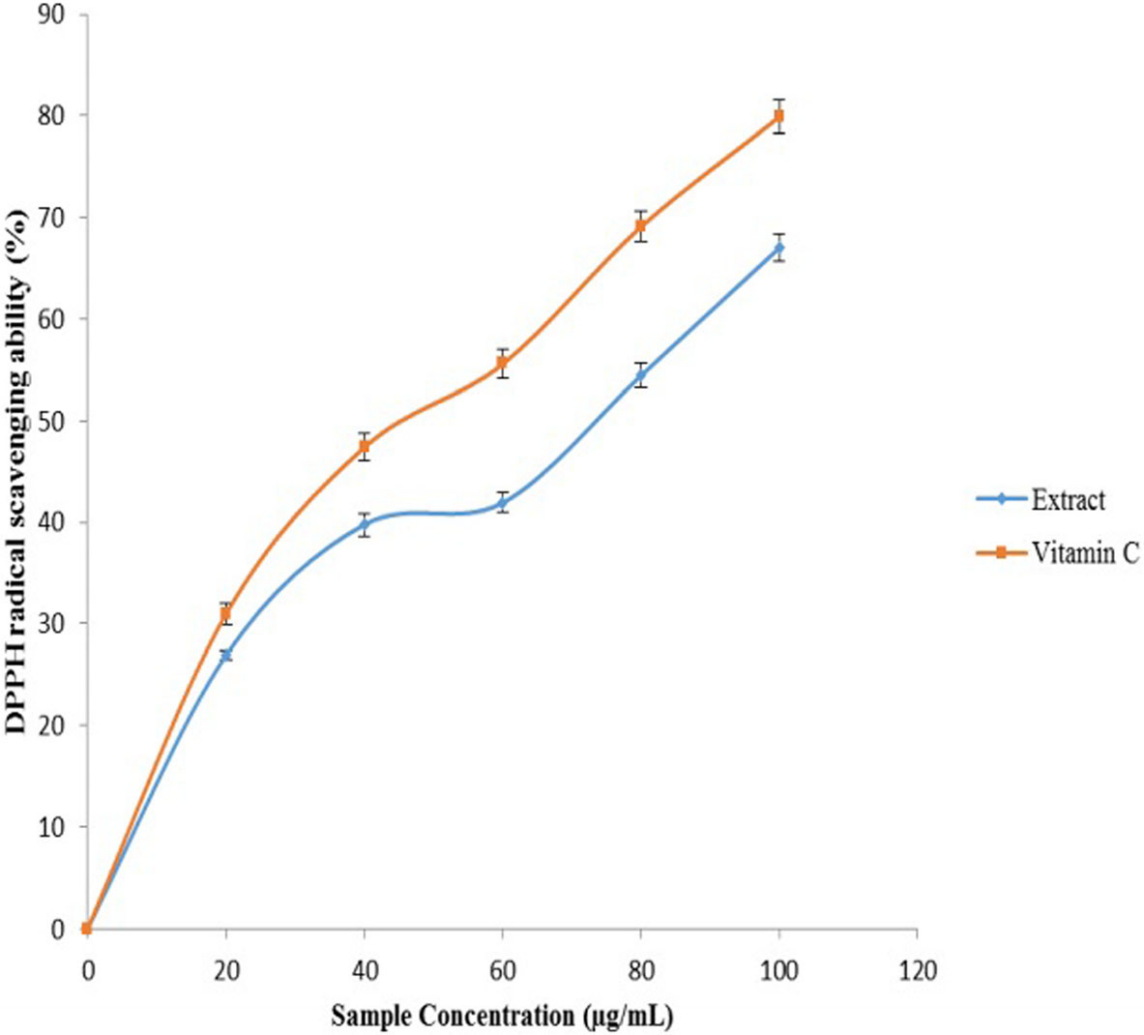
DPPH radical scavenging ability of aqueous extract of *O. gratissimum* leaves and vitamin C

Figure 5 revealed the ferric reducing ability of the extract. The result revealed that the extracts inhibited ferric reducing power in a concentration-dependent manner. However, vitamin C was able to reduce the Fe^3+^ to Fe^2+^ better than the *O. gratissimum* extract as revealed by their IC_50_ values in Table 1.

**Fig. 6:**
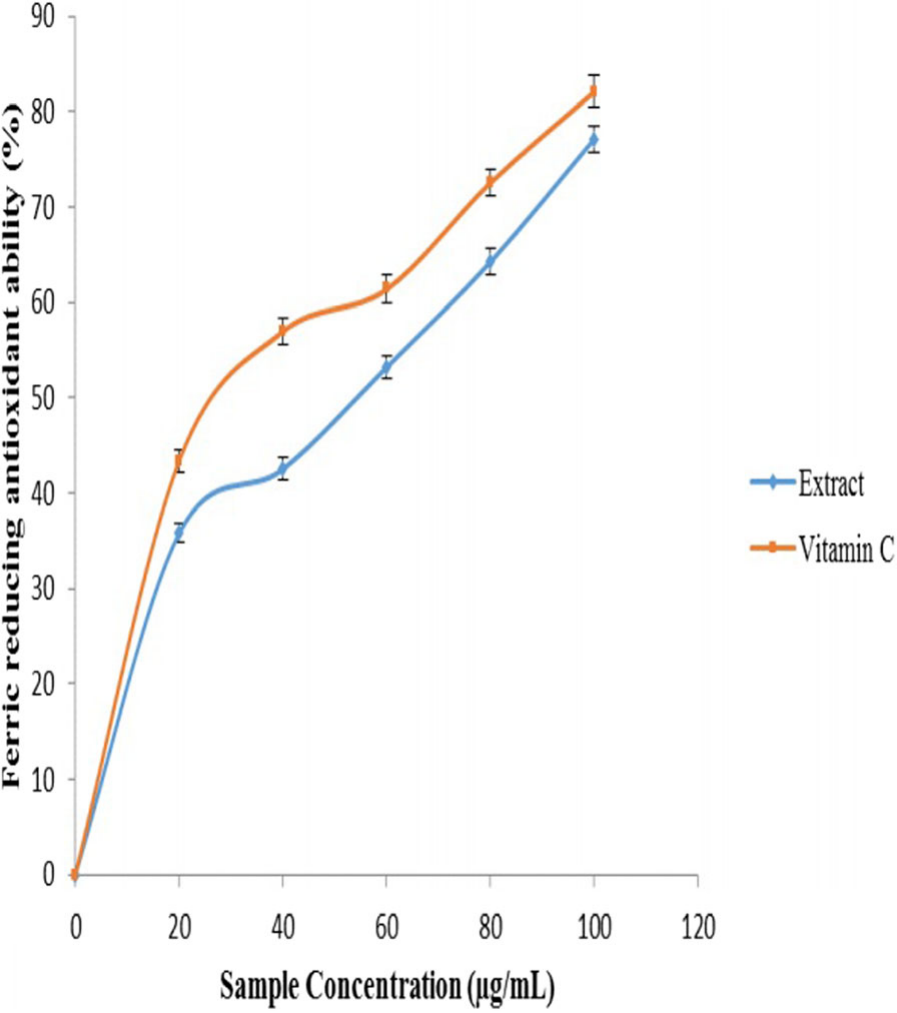
Ferric reducing antioxidant property (FRAP) of aqueous extract of *O. gratissimum* leaves and vitamin C

Fig. 6 revealed the ability of the leaf aqueous extract of *O*. *gratissmum* to scavenge DPPH^.^ in penile and testicular tissues. Aqueous extract of *O*. *gratissmum* was able to scavenge DPPH^.^. However, vitamin C scavenge DPPH^*^ better than the *O. gratissimum* extract as revealed by their IC_50_ values in Table 1.

## Discussion

The mechanisms or actions of cGMP are mainly decreased by the phosphodiesterase-5 enzyme in diverse parts of corpus cavernosum [5]. PDE-5, an important enzyme of the NO/cGMP signaling pathway, that performs a key function in corpus cavernosum weakness, inhibition of nitric oxide-induced cGMP-mediated vasodilation and repairing basal smooth muscle tone and penile detumescence. Thus, the rate of synthesis of cGMP by guanylate cyclase and its degradation by PDEs determines the amount of cGMP within the corpus cavernosum [5]. A protein kinase that decreases cytosolic calcium levels, boosts smooth muscle relaxation leading to penile erection is activated by cGMP [37, 38]. Sildenafil, tadalafil, and vardanafil which are PDE-5 inhibitors do not only boost the level of cGMP [39, 40] but, likewise, stimulate its activation resulting in the increase of NO leading to the penile erection. However, these PDE-5 inhibitor drugs have other negative effects such as nasal congestion, headache, visual aberrations’ dyspepsia, [41]. Furthermore, previous studies have shown that phenolic compounds in plant exhibit PDE-5 inhibition [6, 42, 43]. Hence, the result showed that the extract inhibited PDE-5 activity, this may be due to the presence of phenolic compounds particularly flavonoids, that functions as an endothelium-independent relaxer [33, 44].

Several studies have previously revealed that nitric oxide production is associated with arginase activity regulation [45]. Current examinations have acknowledged arginase in managing sexual disorders [46, 47] leading to upregulation of arginase activity and reduced nitric oxide levels in the penile tissues. In ED, reduced NO are documented owing to improved arginase activities and changed the expression of endothelial NO synthase (eNOS) [48]. Nevertheless, it is important to note that arginase inhibition by the *O. gratissimum* extracts may possibly be an alternative route of increasing the appearance and activities of eNOS, thus raising the bioavailability of nitric oxide. Therefore, the ability of the extract to inhibit an arginase could be due to the presence of phenolics with arginase inhibitory properties which is supported by previous studies [8, 14, 49]. Our findings showed that aqueous extract from *O. gratissimum* inhibited arginase activity. Hence, the inhibitory activity observed could be linked to the presence of flavonoids and phenolic compounds like (−)-rutin, kaempferol, rosmarinic acid, caffeic acid and cichoric acid present in *O. gratissimum* extract reported by [50]. Moreover, Da Silva et al. [51] showed that flavonoids such as rutin are strong inhibitors of arginase enzyme.

Numerous studies have documented that cholinergic nerves in penile tissues must be able to release acetylcholine for the activation of NO production from L-arginine by nitric oxide synthase for better erectile function [7, 52–54]. The inhibition of the enzyme in penile and testicular tissue homogenates suggests that *O. gratissimum* could be a promising plant with beneficial potentials for the management of ED. Thus, inhibition of AChE by the extracts could be linked to the presence of flavonoids and phenolic compounds, which correlates with earlier studies that phenolic-rich plants could inhibit AChE [8, 9, 55, 56].

Angiotensin-II has been associated with hypertension a key causative agent of erectile dysfunction [8, 57]. Hence, the inhibition of ACE leading to the prevention of angiotensin- II synthesis may be a remedial target in the management of ED. Jin [58] documented that the considerable increase in NADPH oxidase activity, production of ROS and inhibition of nitric oxide synthase activity in penile and testicular tissues may be possible due to the elevated levels of angiotensin-II. Thus, hindering the production of angiotensin II could be helpful in the treatment of ED. Hence, these interactions could be due to the phenolic and flavonoids present in the leaves and disulphide bridge of the enzyme [57, 59, 60]. Moreover, flavonoids and phenols such as rutin, kaempferol, cichoric acid and caffeic acid present in the plant have been reported to be strong inhibitors of ACE activity [61, 62], and may be accounted for the greater inhibitory effect displayed by *O. gratissimum*. Thus, this study showed that aqueous extract from *O. gratissimum* inhibited ACE activity, reducing angiotensin II levels in erection organs.

Antioxidants achieve their protective role mainly by hindering the generation of radicals or by scavenging radical’s production [21, 23, 63]. The mechanism of actions acknowledged influencing ferric reducing power as an antioxidant system is via proton transfer and electron transfer [21, 23, 64]. The reducing capacity of the extracts could also indicate potential antioxidant activities [65]. This reducing property could be because of the presence of polyphenols. Reducing power of *O. gratissimum* might have a therapeutic effect on erectile dysfunction.

In this study, *O. gratissimum* at the concentration tested revealed considerably ability to act as radical scavengers. This study revealed that aqueous extracts of *O. gratissimum* scavenged free radicals. The greater scavenging ability of the *O. gratissimum* may be accredited to the presence of secondary metabolites like phenolic acids and flavonoids [66]. All these radicals are involved in the pathogenesis of several disorders including erectile dysfunction. Hence, findings from this investigation shown that *O. gratissimum* extracts possessed antioxidant activity having reasonable scavenging activity towards the various forms of radicals studied [67, 68].

## Conclusion

This study revealed that *O. gratissimum* extracts exhibited inhibitory properties on enzyme activities involved in erectile function and displayed free radical scavenging abilities which might be connected to their phenolic and flavonoids components. This indicates the erection stimulating properties of the plants and possible reasons for its use in the management of erectile dysfunction.

## Abbreviations

ACE: Angiotensin-I-Converting Enzyme
AChE: Acetylcholinesterase
cAMP: Cyclic adenosine monophosphate
cGMP: Cyclic guanosine monophosphate
DPPH: 1,1-diphenyl-2-picryl-hydrazil
ED: Erectile dysfunction
eNOS: Endothelial tissue nitric oxide synthase
nNOS: Neuronal endothelial tissue nitric oxide synthase
NO: Nitric oxide
*O. gratissimum*: *Ocimum gratissimum*
ONOO_2_: Peroxynitrite
PDE-5: Phosphodiesterase-5
RAS: Renin–angiotensin system
